# Sensory Acceptability of Dual-Fortified Milled Red and Yellow Lentil (*Lens culinaris* Medik.) Dal in Bangladesh

**DOI:** 10.3390/foods9080992

**Published:** 2020-07-24

**Authors:** Rajib Podder, Mahmudul Hassan Al Imam, Israt Jahan, Fakir Md Yunus, Mohammad Muhit, Albert Vandenberg

**Affiliations:** 1 College of Agriculture and Bio-resources, The University of Saskatchewan, Agriculture Building 51 Campus Drive, Saskatoon SK S7N 5A8, Canada; bert.vandenberg@usask.ca; 2 CSF Global, Dhaka 1213, Bangladesh; physiomahmud@yahoo.com (M.H.A.I.); arda.jahan89@gmail.com (I.J.); mmuhit@hotmail.com (M.M.); 3 Asian Institute of Disability and Development, University of South Asia, Dhaka 1213, Bangladesh; 4 School of Health, Medical and Applied Sciences, Central Queensland University, Rockhampton, QLD 4701, Australia; 5 College of Pharmacy and Nutrition, The University of Saskatchewan, 104 Clinic Place, Saskatoon, SK S7N 2Z4, Canada; fakir.yunus@usask.ca

**Keywords:** dual fortification, sensory evaluation, iron and zinc deficiency, lentil

## Abstract

This study evaluated the sensory properties of uncooked and cooked milled lentils that were fortified with varying concentrations of Fe and Zn in the form of NaFeEDTA and ZnSO_4_.H_2_O, respectively. Our study was carried out among 196 lentil consumers residing in rural Bangladesh who experience with growing, processing, and marketing lentils. A nine-point hedonic scale was used to rate the appearance, odor, taste, texture and overall acceptability of three uncooked and two cooked lentil (dal) samples made from each of the three milled lentil product types (LPTs), red football, red split and yellow split. Preferences for sensory properties were found to be significantly different among all uncooked lentil samples, but not significantly different for cooked samples, with a few exceptions. This means that the fortification process minimally affects dual-fortified lentil sample (fortified with 16 mg of Fe and 8 mg of Zn per 100 g of lentil), which was compared to another cooked sample (unfortified control), in terms of consumers liking for all four attributes (appearance, odor, taste, and texture).

## 1. Introduction

Iron (Fe) and zinc (Zn) micronutrient deficiencies are two of the most prevalent nutritional threats in the world. About one third and one fifth of the human population are Fe and Zn deficient, respectively [1]. These two micronutrients share common dietary sources and are abundant in the human body [2]. Plant-based diets are becoming popular throughout the world, and legumes such as lentils, chickpeas, dry peas, beans, and fava beans are major dietary sources of protein. Among the legumes, lentils are important for human nutrition because of their relatively high amounts of protein, carbohydrates, and micronutrients compared to some of the staple cereals and root crops [3,4]. More than 50 countries in aggregate produce a global total of about 7.6 Mt of lentils, of which Canada produces about 50% (3.7 Mt) [5]. Lentils contain a substantial amount (dry weight) of protein (25.8 to 27.1%), starch (27.4 to 47.1%), dietary fiber (5.1 to 26.6%) [6,7,8], Fe (73 to 90 mg kg^−1^), Zn (44 to 54 mg kg^−1^), and selenium (425 to 673 µg kg^−1^) [9]. A combination of rice and lentils makes a popular and commonly eaten dish known as “hotchpotch” in many Asian countries, for example, in Bangladesh. This dish provides all essential amino acids, carbohydrates, dietary fiber, and a number of minerals and vitamins. Although lentil has a significant amount of intrinsic Fe and Zn, some antinutritional factors, such as phytate, polyphenols, calcium, and protein can inhibit the absorption of both nutrients from food [9]. The improvement of the concentration of these micronutrients and their bioavailability using a sustainable approach is a prime area for research in order to provide an adequate amount of micronutrients and cope with micronutrient deficiency.

Several organizations are conducting research to improve the micronutrient concentration in crop or food products to cope with global micronutrient deficiency problems. Many approaches are used, including biofortification, food fortification, public health intervention, supplementation, nutrition education, dietary diversification, and food safety measures. These strategies are being employed for various staple crops or foods around the world [10]. In comparison to other approaches, food fortification is now more widely used due to its sustainability for improving the dietary quality of targeted groups or populations rapidly [10,11,12]. Around 84 countries have mandatory fortification programs for various food products based on their existing nutritional status [13]. Several micronutrient-fortified foods/food products are available and are mandatory in the market in different countries, for example, wheat flour in Indonesia, Philippines, Nepal, fortified rice in Papua New Guinea and Costa Rica, maize flour in the USA, soya sauce, salt and edible oil in Bangladesh, milk in Canada and China, etc. [13]. The fortification of pulse crops like lentils or chickpeas is a new research area that began in 2014 at the Crop Development Centre of the University of Saskatchewan, Canada, through the development of Fe-fortified lentils to address Fe-deficiency in humans. A laboratory-scale protocol for fortifying dehulled red lentils with the Fe fortificant NaFeEDTA (ethylenediaminetetraacetic acid iron (iii) sodium salt) was developed [14]. Fortification with 1600 ppm, NaFeEDTA provides 13–14 mg of additional Fe 100^−1^ g in cooked dehulled lentils (dal). An in vitro bioavailability study with Fe-fortified lentils showed that dehulled lentil dal fortified with 28 mg of Fe 100^−1^ g of lentils increased Fe bioavailability to 79% and reduced phytic acid to 25% [15]. The results from these studies led us to develop dual-fortified lentils with Fe and Zn to address Fe and Zn deficiency.

Lentil fortification with both Fe and Zn could have the potential to simultaneously reduce both Fe and Zn deficiency. In this approach, lentils are enriched with extra Fe and Zn to prevent iron deficiency in humans. In this project, research has been initiated to increase both Fe and Zn concentration and bioavailability through a fortification strategy using a modified technique of a previously developed fortification technique by Podder et al. (2017). Initially, a laboratory-based fortification protocol to develop dual-fortified lentil was established. The protocol included the selection of three lentil product types (LPTs) (dehulled red football (RF), red split (RS), and yellow split (YS)), the identification of appropriate methods of fortification, the selection of suitable dosage of added Fe and Zn, and colorimetric changes over the storage period, as well as the in vitro bioavailability of Fe from the dual-fortified lentils [16]. This report describes the results of a sensory analysis of dual-fortified lentil food products.

Sensory analysis is a multidisciplinary science that covers a wide range of social science areas, ranging from food science to statistics to psychology [17]. By definition, “sensory analysis is the identification, scientific measurement and interpretation of the properties (attributes) of a product as they are perceived through the five senses of sight, smell, taste, touch, and hearing” [18]. It captures unbiased human response to food, which helps stakeholders to identify brand effects [19]. Taste, flavor, appearance, and texture are the major attributes of sensory evaluations of food products. The remarks from consumers provide valuable information that help in the development of recommendations for food scientists or commercial food product developers. The present study was designed to undertake an exploratory sensory evaluation to determine the acceptability of dual-fortified lentils (both uncooked and cooked) among 16 to 65-year-old consumers living in Ishurdi, a northern sub-district of Bangladesh.

Lentils are the most frequently consumed legume in Bangladesh where they are a staple food in the daily diet. Similar to other developing and some developed nations, both Fe and Zn deficiencies are common in the Bangladeshi population. Around one third (30%) of Bangladeshi adolescents are anemic, attributable mostly to Fe deficiency [20]. The 2011–12 National Micronutrient Survey of Bangladesh found that the national prevalence of Zn deficiency was approximately 45%, 52%, and 66% among preschool-age children, slum-dwelling preschool children, and non-pregnant, non-lactating women, respectively [21,22]. The expectation from the current study is that dual-fortified red and yellow cotyledon lentil dal will be equally acceptable to the lentil consumers with respect to taste, odor, appearance, texture, and overall acceptability.

The acceptability of fortified food depends on the fortificant type, dose, chemistry of the food vehicle, and interactions between different fortificants [23]. Fortification may create a metallic taste in foods, generate undesirable flavor due to fat rancidity, develop an unacceptable change in color, and degrade the quality of vitamins (e.g., vitamins A and C, which are important for absorption and utilization of Fe) [24]. The expectation of any fortification program is to contain any undesirable changes in food or food products. An earlier study of consumer-level sensory evaluations of cooked and uncooked Fe-fortified lentils (NaFeEDTA) showed that fortified lentils were well received by consumers compared to both unfortified lentils and those fortified with other Fe fortificants [25]. In this study, we hypothesized that dual fortification has a significant effect on liking for the sensory attributes of dual-fortified lentils. This hypothesis was based on the assumptions that there may be identifiable differences between dual-fortified and non-fortified lentils, and that identifying the differences in sensory properties may have major scientific implications for the food science industry. 

## 2. Materials and Methods

### 2.1. Study Design and Selection of Panelists

We carried out a cross-sectional study between 1 February 2019 and 30 April 2019 at the Regional Agricultural Research Station, Ishurdi, Bangladesh. A group of 196 untrained lentil consumers, aged 16–65 years, participated in the sensory evaluation. A total of 50–100 responses are desirable for sensory evaluation according to the sensory evaluation guidelines of the Institute of Food Technologists’ Sensory Evaluation Data [26].

Panelists were included on the basis of their willingness to participate in the study and their general health. The exclusion criteria were (i) having a fever, cold or, gum inflammation; (ii) taking medicines for cancer, thyroid, neurologic, or psychotropic treatment; (iii) being susceptible to an allergic reaction to lentils, iron or zinc; (iv) pregnancy, (v) having chewed betel leaf with betel nut and tobacco (locally known as paan/jarda) less than an hour before the sensory evaluation. A face-to-face interview technique was adopted since it was the appropriate method for filling in the sensory evaluation data by trained research assistants. With the proposed sensory trials, a preliminary assessment of consumer acceptability was conducted prior to carrying out a large-scale study with consumers.

### 2.2. Preparation of Cooked and Uncooked Lentil Samples for Evaluation

**Figure 1:**
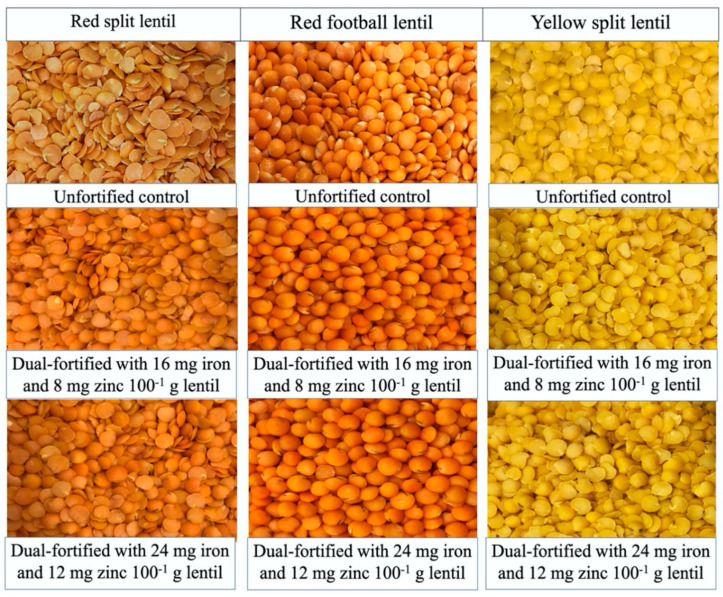
Images of uncooked lentil samples of three lentil product types (red split, left column; red football, middle column; and yellow split, right column), including the unfortified control (**upper row**) and two dual-fortified samples (i) fortified with 16 mg Fe from NaFeEDTA and 8 mg Zn from ZnSO_4_.H_2_O 100^−1^ g of lentil (**middle row**), and (ii) fortified with 24 mg Fe from NaFeEDTA and 12 mg Zn from ZnSO_4_.H_2_O 100^−1^ g of lentil (**lower row**).

**Figure 2:**
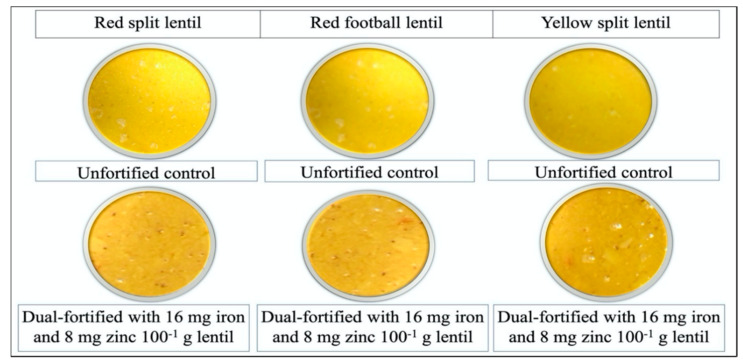
Samples of cooked dal prepared from each of the three product types (red split, left column; red football, middle column; and yellow split, right column) of lentil, including the unfortified controls (**upper three**) and dual-fortified samples (**lower three**) fortified with 16 mg of Fe and 8 mg of Zn 100^−1^ g of lentil.

The most suitable Zn and Fe fortificants were selected after a series of experiments at the University of Saskatchewan Lab [16]. Based on those results, this sensory acceptability study for dual-fortified lentils was conducted. Two dual-fortified uncooked and one unfortified control sample from each of the three milled lentil product types (LPTs) (red football (RF), red split (RS), and yellow split (YS)) were evaluated by the consumers (Figure 1). One randomly selected dual-fortified sample (fortified with 16 mg Fe and 8 mg Zn per 100 g of lentil) and one unfortified control sample from each of the three LPTs were used to prepare a popular traditional recipe [25,27] commonly consumed in Bangladesh (Figure 2). 

Food samples were cooked in the food processing laboratory of the Bangladesh Agricultural Research Institute (BARI), Ishurdi, Pabna, Bangladesh. Hygiene and quality were maintained by using stainless steel cookware to prepare all cooked samples. We prepared a semi-thick lentil soup with each of the 6 different lentil samples. A portion (500 g) of each lentil sample was cooked for about 25 min using a local recipe, i.e., de-ionized water (2.5 L), turmeric (10 g), table salt (20 g), canola oil (30 mL) and chopped onion (100 g). All of the nine uncooked samples were separated into 4-oz white-colored foam cups, labelled with 3-digit codes, for evaluation by individual participants. After completing the uncooked sample evaluation, each participant was given one tablespoon of a cooked lentil dish or lentil soup from each of the six samples separately in 3oz plastic cups labelled with 3-digit codes. Water for rinsing the mouth between tastings was provided to the participants before and after testing each of the dishes.

### 2.3. Data Collection Tools and Techniques

Data were collected at two stages. At the first (screening) stage, a sampling frame was created among the interested participants and we used a simple random sampling technique to finalize the participants. At first, a screening questionnaire was used to collect the information from 200 lentil consumers (aged 16–65 years, who expressed interest in participation) with selected sociodemographic variables. A total of 196 study participants were selected for the final sensory evaluation study. In the second stage, a separate structured questionnaire was used for sensory evaluation. Both questionnaires followed forward–backward translation (English and Bengali). The sensory evaluation form had three parts. Part I covered demographic information, Part II included an evaluation of liking for the appearance, odor, and overall acceptability of the uncooked lentil samples. Similarly, an evaluation of liking for four sensory attributes (appearance, odor, taste, and texture), and the overall acceptability of the cooked dual-fortified lentil samples was also included. Participants’ responses were captured using a 9-point hedonic scale (1 = dislike extremely and 9 = like extremely). In Part III, any opinions/comments from the participants regarding the tasted sample were documented (verbatim), whether they were positive or negative.

Sensory evaluation was carried out in a single day from mid-morning to mid-afternoon. A total of 20 research assistants (RA) were recruited a day before the interviews and were trained by a senior research investigator on the day of evaluation. The training mainly emphasized interview techniques and understanding the sensory evaluation form. After the training session, the data collection team practiced the administration of sensory evaluation forms to ensure the complete understanding and uniformity of the whole data collection process. We organized a total of 20 dual-fortified lentil booths that had uniform white light conditions and furniture for the testing of sensory attributes by the participants. Each participant scored the samples while seated face to face with the research assistant. Twenty participants took a test at one time and the sensory evaluation was conducted in single sessions to avoid reporting bias. Initially, uncooked samples were presented in a white tray for scoring. Then each participant was given one tablespoon of the cooked dishes or lentil soup from each of the samples separately. Deionized water was provided to the participants for oral rinsing before testing the first dish and after testing each of the dishes to cleanse the palate [25,28].

### 2.4. Ethical Considerations

The study was approved by the Research Ethics Office, the University of Saskatchewan, Canada (BH 14–729), the Bangladesh Medical Research Council, Bangladesh (BMRC/NREC/2016-2019/14) and the Asian Institute of Disability and Development (AIDD) Human Research Ethics Committee (HREC) (southasia-hrec-2019-3-01).

The anonymity and confidentiality of the study participants were strictly maintained. Written informed consent was received from each respondent. Unique identification numbers (UID) were assigned to each participant to maintain anonymity and confidentiality. Study participants had the right to withdraw from the study at any time during the interview or sensory evaluation process. No side effects were expected in consideration of the amounts of Fe and Zn fortificants that respondents would consume during the evaluation study. All fortificants were food-grade quality. The toxicity level for Fe in the human body compared to the dose provided was negligible. However, monitoring was undertaken, and an adequate supply of water and necessary precautions were taken before initiating the sensory evaluation. Consent forms were stored separately from the collected data, which was stored on a password-protected computer and all associated computers were also password protected. Hard copies were stored in a locked cabinet. Data will be stored for 5 years after submission of the final report, at which point the soft copies will be deleted from computers and hard copies will be shredded.

### 2.5. HunterLab Colorimetric Measurements of Unfortified and Dual-Fortified Uncooked Lentil Samples and Correlation with Sensory Attributes

The lightness (L*), redness (a*), and yellowness (b*) score of uncooked dual-fortified lentil samples from three LPTs were measured using a HunterLab instrument (Hunter Associates Laboratory Inc., Reston, VA, USA). L* indicates the darkness to lightness, ranging from 0 to 100; a* indicates greenness to redness, ranging from −80 to +80 and b* indicates blueness to yellowness, ranging from −80 to +80 (Wrolstad and Smith, 2010). The HunterLab L*, a* and b* scales were used for measurements three times per sample and the scores were analyzed using ANOVA in SAS 9.4 (SAS Inc. Cary, NC, USA). The sensory data of three attributes (appearance, and overall acceptability) of three LPTs of uncooked lentil samples were correlated with the L*, a*, and b* scores using Pearson’s correlation test.

### 2.6. Statistical Analysis

After data collection was completed, a dataset was prepared in SAS (Statistical Analysis Software, SAS Institute Inc., Cary, NC, USA) version 9.4. Datasets were reviewed by first entering the pretesting questionnaire data as a means of testing the practicability, and to check whether it covered every variable mentioned in the questionnaire. Scores for appearance, odor, taste, texture, and the overall acceptability of the fortified lentils were presented as means with standard deviations (SD). A One-Way Analysis of Variance (ANOVA) was performed to determine mean score differences among food samples, including the control. Statistical significance was set at *p* < 0.05. We tabulated the frequency and percentage as appropriate and used box plots to present sensory data using a 1–9 scale.

### 2.7. Consistency Assessment for Sensory Data Based on Cronbach’s Alpha

Cronbach’s alpha (CA) coefficient was used to measure the consistency of the panelists’ responses since it measures the internal consistency reliability (ICR) of a sensory panel in multi-item evaluation scores [29]. It assessed the measurement error (between zero and one) by squaring correlation (α values) and by subtracting the end results from one, which provides the variation in the error that occurred in the measurement [30,31,32]. The value after subtraction represents the error variance in the score. We assessed the ICR of the liking scores for sensory attributes of 196 panelists in Bangladesh, for the nine uncooked and six cooked samples. Although there is no strict cut-off for CA, several studies report acceptable ICR ranges from 0.70 to 0.95 [33,34].

## 3. Results

### 3.1. Demographic of the Study Participants

The sociodemographic profile of the consumers is presented in Table 1. Among the participants, 59.2% and 40.8% were male and female, respectively, with an age range from 16–65 years, with a major portion (40%) in the 26–35 age range group. In total, 77.7% of the participants were from households where between one and five people were employed. Almost half (48.0%) of the participants had a monthly income ranging between BDT 10,000 and 19,000 (USD ~121–240).

### 3.2. Consumer Attitudes toward Lentil Consumption

Among the participants, 52.0% and 13.8% of the respondents purchased 251–500 g and 751–1000 g of lentils per week, respectively (Table 2). Participants also bought other pulses at lower quantities compared to lentils—46.2% purchased 100–250 g of other pulses (chickpeas, mung beans, black gram, field peas, etc.) weekly, and 38.8% of the participants bought 251–500 g per week. Local markets were the primary source of purchased lentils (89.8%) followed by 8.1% from neighborhood grocery stores. The majority (76.5%) of panelists purchased lentils on a monthly basis and 89.9% preferred to buy red football LPT, followed by 9.7% who preferred red split LPT.

### 3.3. Liking for the Uncooked Fortified Lentil Dal

**Figure 3:**
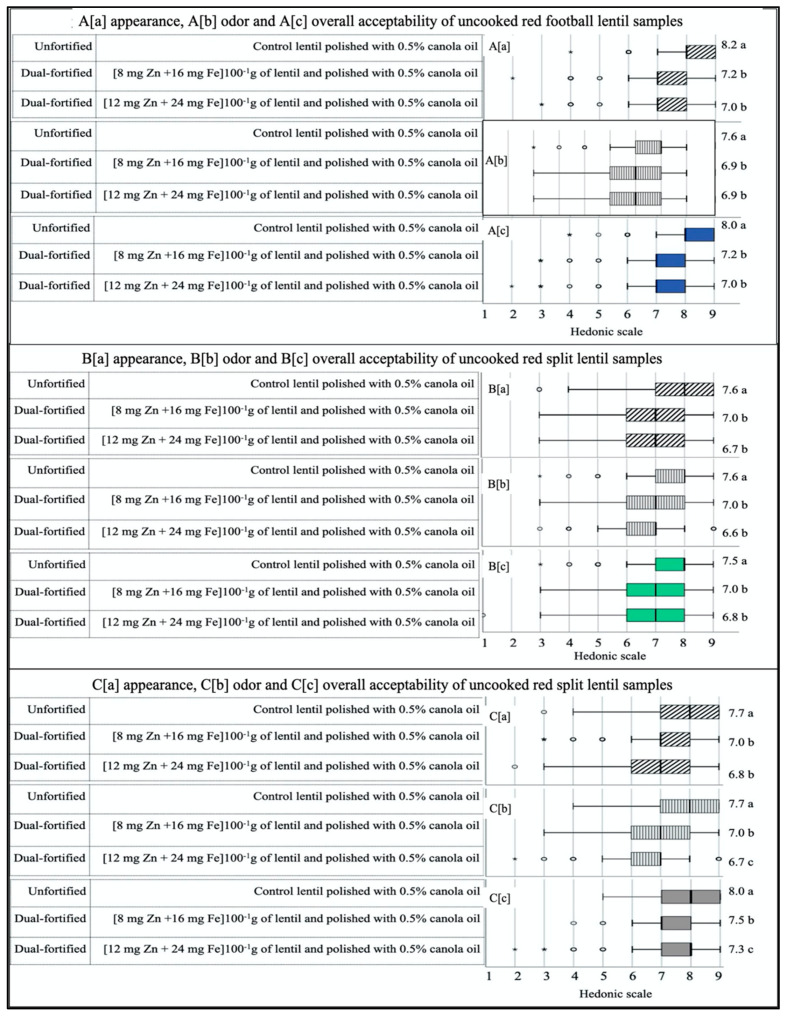
Box plot analysis of hedonic scores (1 = dislike extremely, 9 = like extremely) obtained for three uncooked lentil dal samples (unfortified control lentil polished with 0.5% canola oil; dual-fortified with 8 mg Zn from ZnSO_4_H_2_O +16 mg Fe from NaFeEDTA (100^−1^ g of lentils)); dual fortified with 12 mg Zn from ZnSO_4_H_2_O + 24 mg Fe from NaFeEDTA (100^−1^ g of lentils) from each of the three product types, red football (**A**), red split (**B**) and yellow split (**C**), evaluated for appearance (**A**–**C**(**a**)), odor (**A**–**C**(**b**)), and overall acceptability (**A**–**C**(**c**)), by 196 panelists in Bangladesh. Different letters after mean values in the right column indicated significant differences between three samples within each attribute. Each box plot displays the distribution of data for each sample type separately based on a five-number summary, “minimum”, first quartile (Q1), median, third quartile (Q3), and “maximum”.

Figure 3 show the mean, range, dispersion and outliers of the sensory attributes for the nine uncooked samples. For all three LPTs (RF, RS, and YS), consumer responses varied significantly for appearance, odor, and overall acceptability. The liking scores for sensory attributes, and for overall acceptability were significantly different between control and fortified LPTs for the three samples of both RF and RS lentils; however, insignificant differences were observed within the fortified samples. In YS lentils, the odor and overall acceptability scores significantly varied between fortified and unfortified lentil samples as well as within fortified YS lentil samples. For all attributes and product types, the highest preference score was observed for unfortified control lentil samples, followed by samples fortified with 8 mg Zn from ZnSO_4_H_2_O and 16 mg Fe from NaFeEDTA. The lowest score was recorded for the sample fortified with 12 mg Zn from ZnSO_4_H_2_O and 24 mg Fe from NaFeEDTA.

In general, the box plots for the control samples had a smaller range and less dispersion than those of the two fortified samples for all three LPTs. The box plot skewed either to the right (positive skew) or was neutral for unfortified control, with the average score significantly (*p* < 0.05) higher than that of fortified samples for each of the product types and attributes. In all three LTPs, the mean liking scores for the dual-fortified sample, fortified with 8 mg Zn from ZnSO_4_H_2_O and 16 mg Fe from NaFeEDTA, were significantly (*p* < 0.05) different but closer to the unfortified control compared to the other dual-fortified sample fortified with 12 mg Zn from ZnSO_4_H_2_O and 24 mg Fe from NaFeEDTA.

### 3.4. Liking for the Cooked, Fortified Lentil Dal

**Figure 4:**
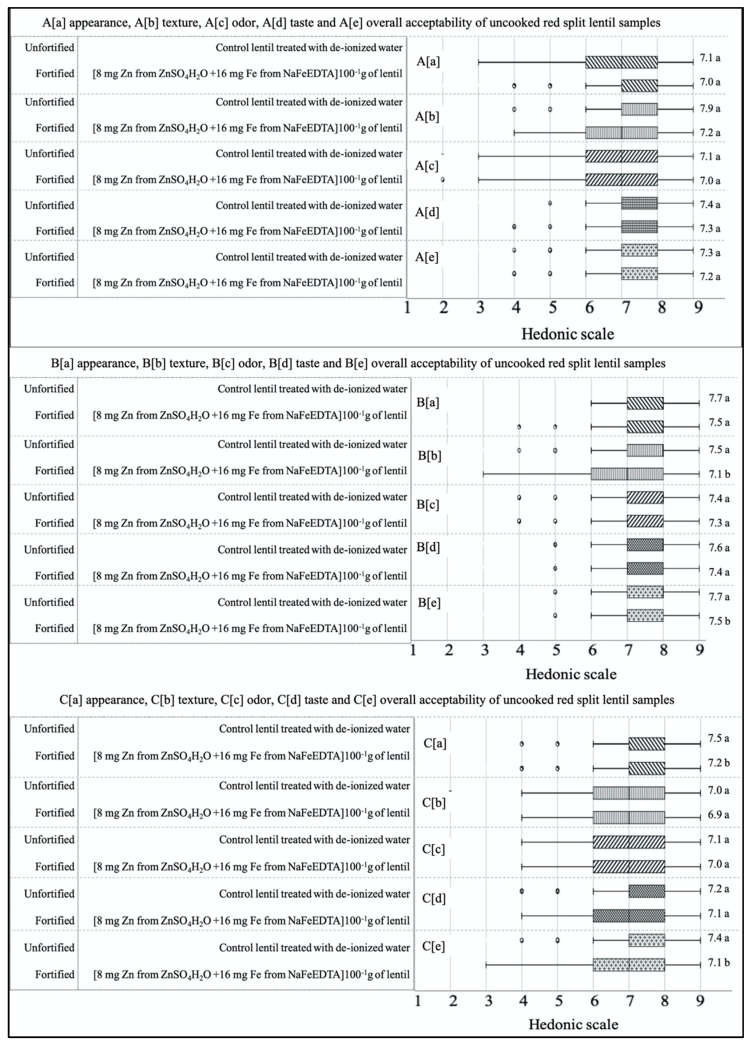
Box plot analysis of hedonic scores (1 = dislike extremely to 9 = like extremely) for two cooked lentil dal samples [unfortified control lentil polished with 0.5% canola oil; dual-fortified with 8 mg Zn from ZnSO_4_H_2_O +16 mg Fe from NaFeEDTA (100^−1^ g of lentils) for each of the three lentil product types—red football (**A**) red split (**B**) and yellow split (**C**). Samples were evaluated for appearance (**A**–**C**(**a**)), texture (**A**–**C**(**b**)), odor (**A**–**C**(**c**)), taste ((**A**–**C**)(**d**)), and overall acceptability (**A**–**C**(**e**)), by 196 panelists in Bangladesh. Different letters after mean values in the right column indicate significant differences between two samples within each attribute. Each box plot displayed the distribution of data for each sample type separately based on a five-number summary, “minimum”, first quartile (Q1), median, third quartile (Q3), and “maximum”.

For all three LPTs, unfortified cooked control samples received the highest mean score for all five attributes (appearance, odor, taste, texture, and overall acceptability) compared to the fortified samples (fortified with 16 mg Fe from NaFeEDTA and 8 mg Zn from ZnSO_4_H_2_O) (Figure 4). An insignificant variation was observed for the two cooked lentil dal samples from all three LPTs evaluated by panelists, except for texture and overall acceptability of RF, and for appearance and overall acceptability of YS lentils. The numerical differences between scores across all samples of each of the three LPTs were very low for all five attributes. Specifically, the box plots for cooked samples showed less dispersion and a narrower range of liking scores for all attributes compared to those for the uncooked samples. All samples scored well (~7.0 = like moderately) for all five attributes.

### 3.5. HunterLab Colorimetric Measurements of Unfortified and Dual-Fortified Uncooked Lentil Samples and Correlation with Sensory Attributes

The results of the lightness (L*), redness (a*) and yellowness (b*) scores of unfortified and dual-fortified lentil samples from three LPTs are shown in Table 3. For all three LPTs, a significant variation was observed between control and dual-fortified lentil samples for all L, a* and b* scores. Again, in all three LPTs, the highest and lowest L, a* and b* values were observed in unfortified-control and dual-fortified samples fortified with 24 mg Fe and 12 mg of Zn 100^−1^ g of lentils. Among the two red football and red split dual-fortified samples, insignificant differences were observed for the L value, but for a* and b* values there were significant differences. Non-significant differences were observed between two dual-fortified samples for all thee scales.

The correlation coefficients between L, a*, and b* scores obtained from HunterLab and sensory acceptability scores were significant at *p* < 0.0001 with a range from 0.92 to 0.99 (Table 4). In the previous study, when we added different doses of Fe solution, the colorimetric test showed that with the increase in Fe dose, the red color of the lentil also became darker [14]. This result also showed a significant correlation with the sensory evaluations of uncooked samples by panelists. The appearance, odor, and overall acceptability were influenced by the increase or decrease in the Fe doses.

### 3.6. Consistency Assessment for Sensory Data Based on Cronbach’s Alpha

Cronbach’s alpha (CA) was used to evaluate the reliability of the sensory data. It creates a “proximity measure between evaluation profiles” by considering both variance and covariance relationships [29]. Table 5 presents the CA scores of both fortified and unfortified cooked and uncooked samples. The CA was ≥ 0.75 for uncooked samples. All the CA scores for cooked samples, except for unfortified YS control lentils polished with 0.5% canola oil, were greater than or equal to 0.80. Mean CA scores for uncooked and cooked samples were 0.84 and 0.81, respectively, which represents a high consistency in the evaluations of all samples using the hedonic scales. 

## 4. Discussion

Sensory evaluation encompasses effective measurements from consumers in terms of their liking, preference, and acceptability of food or food products [35]. The current study was undertaken to understand and evaluate the sensory attributes of dual-fortified lentils among lentil consumers in Bangladesh. The choice of Bangladesh as a study site was made for specific reasons. Lentils are considered a staple or partially staple food in many countries. About 56% of the lentils produced in the world are consumed in Asia [19], with a very high consumption in Bangladesh. Lentils are consumed frequently in daily meals due to their fast cooking properties, and they are also an inexpensive source of protein, carbohydrates, and micronutrients compared to animal sources. This study was conducted in one of the most important lentil-growing regions of Bangladesh. Most farmers of this region have experience with growing, processing, and marketing lentils. Moreover, the national Pulses Research Centre (PRC) of the Bangladesh Agricultural Research Institute (BARI) is located in this region. Several national and international organizations are actively involved with the Bangladesh national health sector in conducting research studies, sensory evaluations, and field trials with fortified foods, e.g., fortified rice. “Daal (pulses), vhat (rice)” or “hotchpotch”, made with pulses (mostly lentils) and rice are common and popular dishes in most South Asian countries, including Bangladesh. Around 60% and 12% of Bangladeshi women consume lentils at a frequency of 3 and 4 days per week, respectively [36]. A similar study reported that 92% of the 384 respondents consumed hotchpotch at least once per week [36]. More than 80% of the lentil dal in the Bangladeshi market is imported from other lentil-growing countries, mostly from Australia and Canada. This provide an enormous opportunity to export dual-fortified lentil products to cope with both Fe and Zn deficiency problems in Bangladesh.

The concept of fortification is emerging in Bangladesh, although few fortified foods are available in the market, and some are under consideration. Two mandatory fortified foods, vegetable oil and salt with vitamin A and iodine, respectively, are now available in Bangladesh [22]. Research studies and evaluations of other fortified food products including rice, lentils, wheat flour, and sugar are underway in Bangladesh. A feasibility study of the field implementation of Fe-fortified lentil with adolescent girls in Bangladesh showed that respondents willingly consumed Fe-fortified lentil meals [22]. A large-scale double-blind community-based randomized controlled trial using Fe-fortified lentils with ~1200 adolescent girls in Bangladesh was recently completed, and results showed a significant effect of Fe-fortified lentils in improving the Fe-status of adolescent girls [37].

In any sensory evaluation study, consumers play a significant role in the preference assessments of product differences and characteristics [38]. The selection of the number of respondents in any consumer test depends on food/food products that need to be evaluated, the purpose of the test, the time frame, and the cost [39]. The recommended sample size for consumer acceptability tests suggest that 50–300 respondents are required for an acceptability test [40]. Suresh and Chandrashekara (2012) described a formula to calculate the sample size and showed that ~96 participants are acceptable to conduct research at the consumer level [41]. In this study, data from 196 participants were used to describe the objectives with statistical significance.

In a sensory analysis at the consumer level, sociodemographic data can be very useful to provide an insight as to whether or not the participants are representative of the total population when a specific food product is evaluated. An earlier study reported that socio-cultural diversity, socio-demographic factors and economic status affect consumer choice regarding functional foods [42]. In the current study, data recorded on participant diversity in terms of age, gender, monthly income, employment status, education, and lentil consumption attitudes confirmed the representativeness of the general consumers (Table 1).

Consumer attitudes toward lentil consumption showed that Bangladeshi consumers preferred red lentil dal compared to other pulses. Among the two product types of red lentil dal, the football type was more preferred (89.8%) than the split type (Table 2). Unlike red lentil dal, dehulled yellow cotyledon lentil dal is usually produced from lentils with green coats, and is not yet well known in the Bangladeshi market. Whole (not decorticated) green lentils have been using in the snack industry for several years, but not for soup preparation at the household level. As lentil demand is increasing around the world [43] and market research for green lentil products has been initiated in different South Asian countries, our goal was to introduce the dehulled yellow lentil dal to the lentil consumers and evaluate consumers’ attitudes to this type of lentil along with the red type. Most of the consumers (around 90%) in Ishurdi bought lentils from the local market, where lentils are sold by scooping from open sacs or in 1–2 kg plastic bags. The previous study [43] in an urban market showed that 37% of consumers bought lentils from local markets or retail shops. This difference could be due to the sociodemographic differences between urban and suburban areas. Fortified lentil is considered a value-added food product that requires packaging in sealed bags to ensure quality and to reduce the risk of adulteration.

Sensory responses to uncooked lentil dal samples revealed significant differences between unfortified and dual-fortified samples for all three LPTs (Figure 3A-C). Although the differences were numerically very low, liking scores from all three attributes (appearance, odor, and overall acceptability) decreased significantly with the increase in Fe and Zn concentration. In all three LPTs, the unfortified controls received higher scores than the fortified samples for all three attributes. Among the three control samples from three LPTs, the RF control got the highest score compared to the other two control samples of RS and YS, indicating the preference for RF lentils compared to RS and YS lentils. Overall acceptability scores for RF, RS, and YS lentils ranged from 7.0 to 8.0, 6.8 to 7.5, and 7.3 to 8.0, respectively. For all the three LPTs, insignificant differences were observed between two dual-fortified lentil samples for all three attributes, except for the order and overall acceptability of YS lentils. A previous study [43] showed that with the increase in Fe fortificants, liking scores decreased in Fe-fortified lentils. The results from this study indicate that Zn fortificants might help to protect the lentil from darkening, even with higher doses of Fe (24 mg of Fe 100^−1^ g of lentil). The results also show that the dual fortification of YS lentils is more susceptible to the development of an off-color appearance than RF and RS lentils with higher doses of Fe and Zn fortificants. In three LPTs, three attributed mean scores of uncooked samples ranged from “like moderately, a score of 7” to “like very much, a score of 8”. Moreover, from all three LPTs and three attributes, several participants scored “9, like extremely” for control samples and a dual-fortified sample fortified with 16 mg of Fe and 8 mg of Zn per 100 g. Overall, the results indicated that dual-fortification with Fe and Zn did not have a large adverse effect on the sensory characteristics of any LPTs.

Non-significant differences were observed between cooked control and dual-fortified lentil samples for all five attributes (appearance, odor, taste, texture and overall acceptability) of the three LPTs, except for texture and the overall acceptability of RF lentils and the appearance and overall acceptability of YS lentils. For all three LPTs, the control lentils received a numerically higher score for all five attributes compared to dual-fortified products. Overall, liking scores for all three LPTs indicated that both cooked samples from each of the three LPTs were accepted equally by the participants. Boxplot comparisons of both uncooked and cooked samples showed that some outlier scores might have greatly influenced the average score of the lentil samples. Some consumers scored the uncooked dual-fortified sample (fortified with 24 and 12 mg of Fe and Zn, respectively) with the two lowermost hedonic scores (dislike extremely, a score of one; dislike very much, a score of two). Some consumers also noted the floating of a black-colored substance, and black spots in the cooked and uncooked samples, respectively. The black spot is the micropylar region of dehulled lentils that is insoluble in water and, after cooking, this region detaches from the cotyledon and floats in the soup. During fortification, this whitish embryonic tissue absorbs fortificant from the solution, resulting in a slight discoloration caused by oxidation [25]. This dark micropylar region could, however, be used as an indicator to help consumers distinguish fortified lentils from unfortified lentils.

In this study, two samples from each of the three LPTs were selected for evaluation by consumers, including one control and one dual-fortified sample with 16 mg and 8 mg of Fe and Zn, respectively. A previous study [16] showed that dual-fortified RF, RS, and YS lentil products fortified with 16 mg Fe and 8 mg Zn per 100^−1^ g of lentils, can provide Fe and Zn at 27.1 to 13.9 mg, 28.0 to 13.4 mg, and 29.9 and 12.1 mg per 100^−1^ g of lentil, respectively. The control samples from each of the three LPTs contain Fe and Zn at 7.5 to 4.3, 7.1 to 4.4 and 5.9 to 3.9 mg per 100^−1^ g of lentil. Each of the 196 participants consumed 15 mg of lentils from each of the cooked samples. Each of the participants consumed a total of 90 g of lentils from both fortified and control lentil samples. From 90 g of lentils, each participant consumed 15.3 mg (4.06 + 4.20 + 4.5 + 1.1 + 1.1 + 0.89) of Fe and 7.76 mg (2.08 + 2.01 + 1.82 + 0.64 + 0.63 + 0.58) mg of Zn. The tolerable upper intake level of iron and zinc per day for males and females (19+ years) is 45 mg/day and 40 mg/day, respectively.

Liking scores for all sensory attributes and for overall acceptability from both uncooked and cooked samples showed that consumers scored differentially for similar samples when cooked lentils were compared to uncooked lentils. The wider range of scores observed for uncooked samples was narrowed down after cooking. The reduced score range could be due to cooking the lentils following a traditional lentil soup preparation recipe [27]. Dry turmeric (*Curcuma longa* L.) powder and onion (*Allium cepa* L.) are the two common ingredients used to cook lentils. The yellow color of turmeric would change the soup’s appearance and suppress the darkness of fortified lentils. The pungent smell of onion also has a significant effect on changing odor and taste profiles and can suppress the metallic taste (if any is detectable) of fortified lentils after cooking [25]. Insignificant differences in sensory attributes were also reported for cooked conventional and fortified rice [44]. Iron and Zn from the fortificants may affect the taste. Since we did not measure biological assessments that affect taste, and as this study was conceptualized to capture a real working scenario in the study population, our study cannot address this issue. In addition, the fortified lentils used in this study were produced in the Saskatchewan Food Industry Development Centre, Canada. In Canada, canola oil is commonly used to polish the lentils after dehulling and cleaning to give them a shiny look that increases consumer attraction. In this study, we did not use palm oil or soybean oil to avoid any interaction between the two different oils, which may have altered the taste and odor. Moreover, participants had the recipe explained to them before the sensory evaluation started.

Sensory analysis helps to evaluate products in a relatively short time and at a low cost with representative consumers who consume the identified product and have sensory skills [45]. The effects of dual fortification on the sensory properties of food are highly variable and depend on the Fe and Zn fortificants and food items [23]. In this study, although consumers could easily distinguish the fortified samples from the control, the overall acceptability was more similar when the samples were cooked. The recommended intake of pulses is 50 g/day/person [46] and the estimated average requirement (EARs) of the Fe and Zn is 29.4 mg and 4.9 mg for males and 18.8 mg and 7.0 mg for females, respectively [23]. Consumption of dual-fortified lentils instead of unfortified lentils could be a prime option to provide a sufficient amount of Fe and Zn in a rapid manner in comparison to other micronutrient intervention approaches mentioned by Northop-Clewes (2013) [23].

The consistency of sensory data was assessed by calculating the Cronbach’s alpha (CA) value, which showed that panelists were consistent in scoring both uncooked and cooked samples and that the CA values were within the acceptable range (0.75 to 0.95) [33,34]. Only one LPT sample (YS control, 0.64) was below the suggested range. This could be due to the inconsistent scoring of consumers for this sample. Although YS lentils were introduced to participants before scoring, some participants did not score the YS sample. One study reported that missing values have an effect on the psychometric properties of any test [47]. However, generalizability cannot be explained through this study since data were cross-sectional in nature. We therefore advise caution when interpreting these specific results.

## 5. Conclusions

Overall, dual fortification decreased consumers’ liking for uncooked lentils, but not cooked ones. We also found high acceptability of the dual-fortified red lentils and no major issues related to acceptability were observed for sensory attributes. We estimated that the dual-fortified samples used in a cooked dal preparation for the three lentil product types can provide approximately 14 mg of Fe and 6.5 mg of Zn from 50 g of lentils. This represents a major part of the estimated average requirement (EARs) of Fe and Zn currently recommended by the World Health Organization (WHO).

## Figures and Tables

**Table 1:** Socio-demographic profile of consumers who participated in the dual-fortified lentil sensory evaluation study in Bangladesh.

Profile Characteristics		Number (%)
Sex	Male	115 (59.2)
	Female	80 (40.8)
Age (years)	16–25	40 (20.9)
	26–35	63 (33)
	36–45	42 (22)
	46–55	37 (19.4)
	56–65	9 (4.7)
Number of employed people in household	1–5	146 (77.7)
	6–10	41 (21.8)
	≥11	1 (0.5)
Total monthly income from all sources (Bangladeshi Taka)	5000–9999 (~90 to 120 USD)	19 (9.7)
	10,000–19,999 (~121 to 240 USD)	94 (48.0)
	20,000–29,999 (~241 to 360 USD)	38 (19.4)
	30,000–39,999 (~361 to 480 USD)	24 (12.2)
	≥40,000 (≥480 USD)	18 (9.2)
Education	Illiterate	3 (1.5)
	Elementary (primary; grade −5) incomplete	11 (5.6)
	Elementary passed	72 (36.9)
	Secondary (grade 10) School Certificate passed	45 (23.1)
	Higher Secondary (grade 12) Certificate passed	64 (32.8)

**Table 2:** Consumer habits and patterns of lentil consumption.

Observation	Consumer Pulse Purchases (g/Family/Week)	Number of Consumers (%)
Lentil purchases	100–250	35 (17.9)
	251–500	102 (52.3)
	501–750	20 (10.3)
	751–1000	27 (13.8)
	≥1001	11 (5.6)
Other pulse purchases (chickpeas, mung beans, black gram, field peas, etc.)	100–250	91 (46.2)
	251–500	76 (38.8)
	501–750	8 (4.1)
	751–1000	7 (3.6)
	≥1001	14 (7.1)
Lentil purchase source	Retail shops	176 (89.8)
	Wholesale	16 (8.1)
	Do not buy or produce	4 (2.0)
Frequency of lentil purchase	Several days in a week	16 (8.6)
	Weekly	18 (9.2)
	Fortnightly	11 (5.6)
	Monthly	150 (76.5)
Lentil product preference market?	Dehulled football	176 (89.8)
	Dehulled split	19 (9.7)

**Table 3:** Lightness (L*), redness (a*) and yellowness (b*) scores of one unfortified and two dual-fortified dehulled red football, red split and yellow split lentil samples prepared using Fe and Zn from NaFeEDTA and ZnSO_4_H_2_O, respectively.

Samples	Fortificant Dose/s Added/100 g of Lentil		CIELAB Color Score ^a^		
	Fe (mg) from NaFeEDTA	Zn (mg) from ZnSO_4_H_2_O	Lightness (L)	Redness (a*)	Yellowness (b*)
Red football					
Sample 1 ^b^	Unfortified and polished ^d^		52.4 ± 0.1 a	31.7 ± 0.1 a	46.2 ± 0.1 a
Sample 2 ^c^	16	8	50.8 ± 0.2 b	28.2 ± 0.2 b	40.6 ± 0.1 b
Sample 3 ^c^	24	12	50.8 ± 0.3 b	27.8 ± 0.1 c	39.7 ± 0.2 c
Red split					
Sample 1 ^b^	Unfortified and polished ^d^		55.1 ± 0.3 a	31.4 ± 0.3 a	46.6 ± 0.4 a
Sample 2 ^c^	16	8	53.3 ± 0.1 b	28.9 ± 0.2 b	43.4 ± 0.2 b
Sample 3 ^c^	24	12	53.2 ± 0.2 b	27.0 ± 0.0 c	41.4 ± 0.1 c
Yellow split					
Sample 1 ^b^	Unfortified and polished ^d^		62.1 ± 0.2 a	12.5 ± 0.1 a	50.9 ± 0.1 a
Sample 2 ^c^	16	8	59.5 ± 0.2 b	10.6 ± 0.3 b	45.6 ± 0.3 b
Sample 3 ^c^	24	12	59.2 ± 0.3 b	10.6 ± 0.1 b	45.8 ± 0.3 bc

^a^ Mean ± SD. Mean scores for lightness (L*), redness (a*) and yellowness (b*) score followed by different Roman letters within columns are significantly different (*p* < 0.0001). ^b^ Unfortified control lentil; ^c^ Dual-fortified lentil with NaFeEDTA and ZnSO_4_H_2_O; ^d^ polished with 0.5% canola oil.

**Table 4:** Correlation coefficients between colorimetric data lightness (L*), redness (a*) and yellowness (b*) score obtained from HunterLab and sensory acceptability scores from Bangladeshi consumers for three attributes (appearance, and overall acceptability) of each of three uncooked product types (red football, red split and yellow split) of lentil samples. all the correlation coefficients were found significant at *p* < 0.0001.

Sensory Attributes	Lentil Product Types								
	Red Football			Red Split			Yellow Split		
	L	a*	b*	L	a*	b*	L	a*	b*
Appearance (n = 3)	0.99	0.99	0.99	0.96	0.99	0.99	0.99	0.99	0.98
Overall acceptability (n = 3)	0.99	0.99	0.99	0.98	0.98	0.99	0.93	0.95	0.97

L*, Lightness; a*, redness; b*, yellowness.

**Table 5:** Internal consistency reliability (CA) of the sensory panelists’ ratings of uncooked red football, red split and yellow split lentil and cooked dal samples.

Uncooked Samples			CA Score
Red football	Unfortified	Control lentil polished with 0.5% canola oil	0.88
	Fortified	[8 mg Zn +16 mg Fe]100^−1^ g of lentil and polished with 0.5% canola oil	0.87
	Fortified	[12 mg Zn + 24 mg Fe]100^−1^ g of lentil and polished with 0.5% canola oil	0.86
Red split	Unfortified	Control lentil polished with 0.5% canola oil	0.81
	Fortified	[8 mg Zn +16 mg Fe]100^−1^ g of lentil and polished with 0.5% canola oil	0.75
	Fortified	[12 mg Zn + 24 mg Fe]100^−1^ g of lentil and polished with 0.5% canola oil	0.81
Yellow split	Unfortified	Control lentil polished with 0.5% canola oil	0.89
	Fortified	[8 mg Zn +16 mg Fe]100^−1^ g of lentil and polished with 0.5% canola oil	0.81
	Fortified	[12 mg Zn + 24 mg Fe]100^−1^ g of lentil and polished with 0.5% canola oil	0.87
Average value for all the uncooked samples			0.84
Cooked samples			
Red Football	Unfortified	Control lentil polished with 0.5% canola oil	0.80
	Fortified	[8 mg Zn +16 mg Fe]100^−1^ g of lentil and polished with 0.5% canola oil	0.81
Red split	Unfortified	Control lentil polished with 0.5% canola oil	0.83
	Fortified	[8 mg Zn +16 mg Fe]100^−1^ g of lentil and polished with 0.5% canola oil	0.87
Yellow split	Unfortified	Control lentil polished with 0.5% canola oil	0.64
	Fortified	[8 mg Zn +16 mg Fe]100^−1^ g of lentil and polished with 0.5% canola oil	0.89
Average value for all the cooked samples			0.81
